# Supplementary material for: Patterns of Intron Gain and Loss in Fungi
Source: PLoS Biol. 2004 Nov 30;2(12):e422. doi: 10.1371/journal.pbio.0020422 (PMC532390; doi:10.1371/journal.pbio.0020422)
Supplement: Table S1 — Also available at http://genes.mit.edu/NielsenEtAl/. (4.3 MB ZIP). [file pbio.0020422.st001.zip › NielsenEtAl/html/947.html]

AN6900.1.NCU07550.1.MG08905.1.FG06702.1


```
 CLUSTAL W (1.82) Multiple Sequence Alignments - Introns Inserted


Sequence 1: MG08905.1	231 aa
Sequence 2: FG06702.1	247 aa
Sequence 3: NCU07550.1	248 aa
Sequence 4: AN6900.1	249 aa
Alignment Length: 251 aa
Number Identitical Residues: 117 aa
Alignment Score (without introns) 5916


MG08905.1 	MARKFFVGGNFKI2FTS-------------------SE~VVISPPHVYLSHTRSLAKK-E
NCU07550.1	MARKFFVGGNFKM2NGTRKDLKAIVDNLNNAQLDPNAE1VVIAPPALYLDFVKQNLQKPN
FG06702.1 	MARKFFVGGNFKM2NGSKSSIKEIVENLNNADLDKNAE1VVVSPPALYLPLVRETLRK-E
AN6900.1  	MPRKFFVGGNFKM2NGNAESTTSIIKNLNSANLDKSVE~VVVSPPALYLLQAREVANK-E
          	*.**********:   . .. .   .. ..:. . . * **::** :**  .:.  .* :

MG08905.1 	IEVAAQNVFDKADGAYTGETSVSQLKDLGINWVILGHSERRTILGESDE~VVASKTKYAI
NCU07550.1	VEVAAQNVFNKPNGAFTGEISATQLLDLGVKWVILGHSERRNELGESDE~FIASKTKYAL
FG06702.1 	IEVAAQNVYDKPNGAFTGEISVSQLKDSDINWAILGHSERRTIIGESDE~VISSKTKYAT
AN6900.1  	IGVAAQNVFDKPNGAFTGEISVQQLREANIDWTILGHSERRVILKETDE0FIARKTKAAI
          	: ******::*.:**:*** *. ** : .:.*.********  : *:** .:: *** * 

MG08905.1 	DNGLKVIWCCGESLE~ERESDKTVEVVTRQLEALKAKVGTSGWENIVIAYEPIW~AIGTG
NCU07550.1	DNGISVIWCCGESKD~TRQAGETIKFVENQLAALAKEIN--DWKNVVIAYEPIW~AIGTG
FG06702.1 	ENGLKVIWCCGESLE~TREAGKTIEFVSAQIESLKSQI--SDWSNIVIAYEPIW~AIGTG
AN6900.1  	EGGLQVIFCIGETLE0EREANKTIDVVTRQLNAAAKELSKEQWAKVVIAYEPVW2AIGTG
          	:.*:.**:* **: :  *::.:*:..*  *: :   ::... * ::******:* *****

MG08905.1 	KVATPEQAQDAHAAIRGYLKSKVSDKVAEETRILYGGSVNEKNCGELSQKPDIDGFLVGG
NCU07550.1	LVATKEQAQEVHAAIRSWLKQNVSDKVAEETRILYGGSVNAKNCKDLAKEQDIDGFLVGG
FG06702.1 	KVATTEQAQEVHKAIRDLLRS-ISDKVADETRILYGGSVNEKNCGELSKQADIDGFLVGG
AN6900.1  	KVATTEQAQEVHSAIRKWLKDAISAEAAENTRIIYGGSVSEKNCKDLAKEADIDGFLVGG
          	 *** ****:.* ***  *:. :* :.*::***:*****. *** :*::: *********

MG08905.1 	ASLKPAF1VDICTCKSA
NCU07550.1	ASLKPEF1VDIINANL-
FG06702.1 	ASLKPAF1VDIINATKQ
AN6900.1  	ASLKPAF1VDIVNARL-
          	***** * *** ..
```
